# Data on energy, exergy analysis and optimisation for a sugar factory^☆^

**DOI:** 10.1016/j.dib.2015.09.028

**Published:** 2015-10-03

**Authors:** Tolga Taner, Mecit Sivrioglu

**Affiliations:** a Department of Motor Vehicles and Transportation Technology, Vocational School of Technical Sciences, Aksaray University, 68100 Aksaray, Turkey; b Department of Mechanical Engineering, Engineering Faculty, Gazi University, 06570 Ankara, Turkey

**Keywords:** Energy, Exergy, Raw, Energy quality, Data

## Abstract

A huge amount of energy is consumed during sugar production in the food industry. The large amount of steam used and the power of the turbine power plant are key factors. This makes energy and exergy analysis important in sugar factories. The data given in the following paper are related to input and output information of the paper entitled Energy – exergy analysis and optimisation of a model sugar factory in Turkey by Taner and Sivrioglu (2015) [1]. Factory total energy efficiency and exergy efficiency are found to be *η*_enT_=72.2% and *η*_exT_=37.4%, respectively, and according to these results, the total energy quality *∅*_T_=0.64. These results indicate higher efficiency than similar studies (Vuckovic et al., 2014; Pellegrini and Oliviera Junior, 2011; Deshmukh et al., 2013; Palacios-Bereche et al., 2015) [2], [3], [4], [5]. This study can be a model for these similar factories by Taner and Sivrioglu (2015) [1].

**Specifications table**

**Table:** 

Subject area	*Mechanical Engineering (Thermodynamic)*
More specific subject area	*Energy & exergy*
Type of data	*Tables*
How data was acquired	*Technical data sheet, analysed and processed output data*
Data format	*Raw, analysed (xls)*
Experimental factors	*Mass, temperature and pressure inputs/outputs*
Experimental features	*Inputs mass and output production from factory processes were taken from the monitor centre during the year (2012–2013).*
Data source location	*Konya, Turkey*
Data accessibility	*Data is provided in Supplementary materials directly with this article*

**Value of the data**

• We describe a methodology to obtain energy and exergy efficiencies for a model of sugar factory.
• We improve a general model of a sugar production process based on data provided by a genuine plant.
• The efficiencies calculated in this study can be used for benchmarking of similar factories.

## 1 Data

The integrated sugar factory׳s name is Çumra Sugar Integrated Plant, Turkey. The process data were taken with permission from the factory manager. These data were recently taken in collaboration with the Department of Factory Central Monitoring and Directorate of Maintenance and Energy (Year 2011 to 2013) [1]. A huge amount of energy is consumed during sugar production in the food industry. Calculations and analyses are performed from the point of view of energy and exergy efficiency [1].

## 2 Experimental design, materials and methods

To define the relationship between the calculated energy and exergy analysis results, Linear Regression Analysis from the Statistical Methods is applied with the SPSS 17.0 software package. The effect of the integration of Solid Oxide Fuel Cell (SOFC) technology was performed in a sugar–ethanol factory [6]. A thermal integration technique was used another study [7]. The thermodynamic performance of the plant was reached to assess [8]. In this study, these similar subjects were discussed in our research.

The data in this data article has been generated by following these main steps:

1. Method and equations regarding energy and exergy analysis.
2. Factory energy and exergy analysis results.

This method allowed the identification of measures able to optimise the energy performance of the sugar factory. The optimisation tool, with the use of computational procedures developed in Excel programme, has been used in order to calculate the energy and exergy analysis. These values are also calculated by using the thermodynamics laws and other steam tables and then verified.

Energy and exergy analysis results of the raw juice production process data are seen in Table 2 in Ref. [1]. According to these, net energy transfer, exergy, efficiencies and energy quality of the raw juice production process are found to be En_net2_=333.17 [kJ/pg], Ex_ℓ2_=268.03 [kJ/pg], *η*_en2_=98.1%, *η*_ex2_=68.6% and *∅*_2_=0.80, respectively [1]. Energy and exergy analysis results of the raw juice purification process data are seen in Table 3 in Ref. [1]. According to these, the net energy transfer, exergy, efficiencies and energy quality of the raw juice purification process are found to be En_net3_=13,690.1 [kJ/pg], Ex_ℓ3_=480.0 [kJ/pg], *η*_en3_=85.1%, *η*_ex3_=89.8% and *∅*_3_=0.04, respectively [1]. A low quality of energy stems from the basic processes in the production of sugar in this process. Energy and exergy analysis results of the raw juice stiffening process data are seen in Table 4 in Ref. [1]. According to these, the net energy transfer, exergy, efficiencies and energy quality of the raw juice stiffening process are found to be En_net4_=14,352.6 [kJ/pg], Ex_ℓ4_=7564.2 [kJ/pg], *η*_en4_=86.7%, *η*_ex4_=61.7% and *∅*_4_=0.53, respectively [1]. Energy and exergy analysis results of the thick juice refining process data are seen in Table 5 in Ref. [1]. According to these, the net energy transfer, exergy, efficiencies and energy quality of the thick juice refining process are found to be En_net5_=5708.0 [kJ/pg], Ex_ℓ5_=3695.1 [kJ/pg], *η*_en5_=87.7% and *η*_ex5_=48.6% and *∅*_5_=0.65, respectively [1]. Energy and exergy analysis results of wet crystal sugar drying (granulation) and cooling process data are seen in Table 6 in Ref. [1]. According to these, the net energy transfer, exergy, efficiencies and energy quality of the wet crystal sugar drying (granulation) and cooling process are found to be En_net6_=1,459.4 [kJ/pg], Ex_ℓ6_=175.2 [kJ/pg], *η*_en6_=78.3%, *η*_ex6_=73.7% and *∅*_6_=0.12, respectively [1]. Energy and exergy analysis results of factory energy generation process data are seen in Table 7 in Ref. [1]. According to these, the net energy transfer, exergy, efficiencies and energy quality of the factory energy generation process are found to be En_net7_=82,314.5 [kJ/pg], Ex_ℓ7_=63,397.3 [kJ/pg], *η*_en7_=46.4%, *η*_ex7_=27.7% and *∅*_7_=0.77, respectively [1].

Energy and exergy analysis results of a factory general process are seen in Table 8 in Ref. [1]. According to these results, the amount of factory total net energy transfer En_T_=117,857.8 [kJ/pg] and total exergy loss Ex_T_=75,579.8 [kJ/pg]. Factory total energy efficiency and exergy efficiency are found to be *η*_enT_=72.2% and *η*_exT_=37.4%, respectively, and according to these results, the total energy quality *∅*_T_=0.64 [1].
