# Supplementary material for: Data on energy, exergy analysis and optimisation for a sugar factory
Source: Data Brief. 2015 Oct 3;5:408–10. doi: 10.1016/j.dib.2015.09.028 (PMC4773381; doi:10.1016/j.dib.2015.09.028)
Supplement: Supplementary file 2 — Supplementary material [file mmc2.pdf]

CONFLICT OF INTEREST FORM

Manuscript Title: Data of energy, exergy analysis and optimisation for a sugar factory

This statement is to certify that all Authors have seen and approved the manuscript being submitted. We warrant that the article is the Authors' original work. We warrant that the article has not received prior publication and is not under consideration for publication elsewhere. On behalf of all Co-Authors, the corresponding Author shall bear full responsibility for the submission. This research has not been submitted for publication nor has it been published in whole or in part elsewhere. We attest to the fact that all Authors listed on the title page have contributed significantly to the work, have read the manuscript, attest to the validity and legitimacy of the data and its interpretation, and agree to its submission to the Journal of Epidemiology and Global Health.

The authors whose names are listed immediately below certify that they have NO affiliations with or involvement in any organization or entity with any financial interest (such as honoraria; educational grants; participation in speakers' bureaus; membership, employment, consultancies, stock ownership, or other equity interest; and expert testimony or patent-licensing arrangements), or non-financial interest (such as personal or professional relationships, affiliations, knowledge or beliefs) in the subject matter or materials discussed in this manuscript.

**Author Names**	**Signatures**
Tolga Taner, PhD (Corresponding Author)	
Mecit Sivrioglu, PhD (Co-Author)	

Date: 19/09/2015
